# The Role of Non-Coding RNAs as Prognostic Factor, Predictor of Drug Response or Resistance and Pharmacological Targets, in the Cutaneous Squamous Cell Carcinoma

**DOI:** 10.3390/cancers12092552

**Published:** 2020-09-08

**Authors:** Marianna Garofoli, Mariateresa Volpicella, Michele Guida, Letizia Porcelli, Amalia Azzariti

**Affiliations:** 1 Experimental Pharmacology Laboratory, IRCCS IstitutoTumori Giovanni Paolo II, 70124 Bari, Italy; m.garofoli@oncologico.bari.it (M.G.); l.porcelli@oncologico.bari.it (L.P.); 2 Department of Biosciences, Biotechnologies and Biopharmaceutics, University of Bari, 70126 Bari, Italy; mariateresa.volpicella@uniba.it; 3 Rare Tumors and Melanoma Unit, IRCCS IstitutoTumori Giovanni Paolo II, 70124 Bari, Italy; m.guida@oncologico.bari.it

**Keywords:** cutaneous squamous cell carcinoma, non-coding RNA, microRNA, circularRNA, long non-coding RNA

## Abstract

**Simple Summary:**

Cutaneous Squamous Cell Carcinoma is poorly understood at a molecular level, however emerging evidence have highlighted that non-coding RNAs play a key role in the oncogenesis and tumor progression. Herein we focused on the translational potential of non-coding RNAs, such as miRNAs, circular RNAs and lncRNAs, by reporting cutting edge findings showing that they can be considered as potential predictors of response to diverse class of drugs, including chemotherapeutics and immune checkpoints inhibitors and as reliable prognosticators. Additionally, by reporting the molecular mechanisms of drug sensitivity and resistance in which non-coding RNAs are involved, we highlighted their potential role as druggable targets and suggested new therapeutic options for CSCC patients.

**Abstract:**

Cutaneous squamous cell carcinoma (CSCC) is the most common keratinocyte-derived skin cancer in the Caucasian population. Exposure to UV radiations (UVRs) represents the main risk carcinogenesis, causing a considerable accumulation of DNA damage in epidermal keratinocytes with an uncontrolled hyperproliferation and tumor development. The limited and rarely durable response of CSCC to the current therapeutic options has led researchers to look for new therapeutic strategies. Recently, the multi-omics approaches have contributed to the identification and prediction of the key role of non-coding RNAs (ncRNAs), such as microRNAs (miRNAs), circularRNAs (circRNAs) and long non-coding RNAs (lncRNAs) in the regulation of several cellular processes in different tumor types, including CSCC. ncRNAs can modulate transcriptional and post-transcriptional events by interacting either with each other or with DNA and proteins, such as transcription factors and RNA-binding proteins. In this review, the implication of ncRNAs in tumorigenesis and their potential role as diagnostic biomarkers and therapeutic targets in human CSCC are reported.

## 1. Introduction

Cutaneous squamous cell carcinoma (CSCC) is the most common non-melanoma skin cancer, accounting for about 20% of all cutaneous malignancies [1]. Besides exposure to ultraviolet radiation (UVR), which is the main risk factor involved in the CSCC etiopathogenesis, other risk factors are old age, immunosuppression, smoking, and genetic factors. UV radiation induces a significant accumulation of DNA damage in cutaneous cells, providing a wide mutational landscape with a ‘UV signature’ in which the high mutation burden is the main actor that drives CSCC carcinogenesis through the uncontrolled hyperproliferation of the epidermal keratinocytes [2,3]. Epidemiological and clinical evidence reveal a higher incidence rate in the Caucasian population, in the elderly due to cumulative exposure to UVR in a lifetime, and in organ transplant recipient (OTRs) patients in which immunosuppressive therapy promotes the development of aggressive CSCC more susceptible to recurrences and metastasis [3,4,5,6].

The main treatment for CSCC is surgical resection and/or radiotherapy, although local recurrences and rarely metastases (1.9–2.6%) can occur. In these cases, and for unresectable CSCC, the chemotherapy options based on cisplatin, 5-fluorouracil, doxorubicin, or bleomycin, as single agents or in combination, have demonstrated limited and unsuccessful efficacy. Therefore, new treatments have been developed to improve the CSCC response to therapy, including the target therapy with the epidermal growth factor receptor (EGFR) inhibitors Cetuximab.

After years of absence of new therapeutic options for CSCC, recently new studies were carried out with immunecheckpoint inhibitors, first of all, the antiPD-1 (programmed cell death protein 1) Pembrolizumab and Cemiplimab. The antiPD-1 Cemiplimab has been recently evaluated in patients with advanced CSCC (study EMPOWER-CSCC-1). The results of the phase 1 and phase 2 studies, not fully mature, show objective responses of 50% with about 10% complete responses, and a one-year survival time of approximately 80% [7,8].

On the bases of these promising results, in September 2018, the US Food and Drug Administration (FDA) approved Cemiplimab therapy for the treatment of patients with metastatic or locally advanced CSCC. The European Medicines Agency (EMA) has also approved this drug with the same therapeutic indication in 2019. From July 2020 it received reimbursement from the Italian national health system. Due to clinical meaningful, durable responses, and acceptable safety, immunotherapy with checkpoint inhibitors is at present the standard of care for patients with recurrent or metastatic CSCC [1,9,10].

Despite the successful results obtained with immune checkpoint inhibitors, about half of the patients do not respond, and many of them become resistant to immunological therapy [7,11], leading to the huge need to identify new clinically useful biomarkers able to increase the treatment efficacy for CSCC. In order to discover novel predictive factors and potential therapeutic targets, able to overcome the driving force of tumorigenesis and the drug resistance in CSCC, the identification of the molecular mechanisms involved in the initiation and progression of the tumor is essential.

In recent years, the Next-Generation Sequencing (NGS) technologies have offered an extraordinary opportunity to identify and measure different kinds of ncRNAs sequences that play a crucial role in the regulation of several cellular processes, such as chromatin remodelling, transcription, post-transcriptional modifications, and signal transduction [12,13,14]. Among them, some are highly conserved, such as miRNAs and circRNAs, while others differ between species, such as lncRNAs. Specifically, the aberrant alterations of ncRNA intracellular networks can lead to the development of pathological conditions, such as tumorigenesis. ncRNAs are indeed involved in the initiation, progression, migration, invasion, and chemoresistance in different cancer types, including CSCC, and modulate the cellular responses by acting as pro- or anti-tumor factors [3,15,16,17,18,19]. Given their implication in tumor pathogenesis, the ncRNAs have been elected as potential biomarkers and therapeutic targets in human CSCC. In this review, we focus on the mutational landscape which drives the tumor transformation of healthy keratinocytes into CSCC cells, and we propose to explore the oncogenic and tumor suppressor role of ncRNAs in CSCC cancerogenesis.

## 2. The Mutational Burden of CSCC

UV radiations represent one of the main damage factors to DNA in animal cells. In the solar spectrum, UVB rays (280–320 nm) represent the most energetic, mutagenic, and carcinogenic radiations for epidermal cells, leading to the formation of two types of DNA damage involved in the development of skin cancer: cyclobutane pyrimidine dimers (CPDs) and pyrimidine (6–4) pyrimidone photoproducts (64PPs), both involved in the development of skin cancer [20,21].

CPDs represent the main class of UVB-induced damages, and among them thymine/cytosine (TC) and cytosine/cytosine (CC) dimers are the most mutagenic. It has been found that these mutations are the most frequent in the TP53 gene of cancer cells. On the other hand, 64PPs seem to be less mutagenic and are efficiently repaired (approximately 90% of 64PPs are repaired at 3 h after irradiation). Both UVB and the less energetic UVA (320–400 nm) radiations can also cause DNA oxidative lesions through the formation of singlet oxygen of purine and pyrimidine bases and the subsequent formation of 8-hydroxydeoxyguanosine (8-OHdG) that induces a G > T transition in the DNA strand [21].

Available data on the efficiency of DNA photoproducts in epidermal cells show that UVR exposure mainly induces the formation of pyrimidine dimers, explaining the “mutational signature” in skin cancers [20].

Whole exome sequencing (WES) analysis has revealed that CSCC is also a tumor with the “UVR mutational signature”, showing a marked mutation burden with about 50 mutations per megabase pair DNA with a prevalence of cytosine > thymine (C > T) transitions at pyrimidine sites that represent the 68% of all somatic mutations induced by UVR damage [2,6,22].

In normal skin, long-term UVR exposure induces a significant accumulation of DNA damage and mutations in epidermal cells that are constantly renewed through proliferation and differentiation processes. Interestingly, significant evidence shows that sun-exposed epidermal cells carry many of the CSCC-specific mutations such as TP53 and NOTCH1/2 mutations, with an incidence rate 10-fold lower than CSCC cells [23]. Probably, the continuous accumulation of UVR-induced mutations and the constant renewal of damaged epidermal cells support the multiple mutational events that lead to the activation of oncogenes or the inactivation of tumor suppression genes with resultant CSCC development [5,20].

As supported by the COSMIC database (Catalogue of Somatic Mutation in Cancer) and the WES analysis performed on CSCC patient-derived samples, the most commonly altered and “potentially targetable” genes in CSCC are involved in cell cycle, proliferation, and squamous cell differentiation pathways and in chromatin remodelling. In Table 1 the most common genes are summarized with higher mutation rates such asTP53, NOTCH1 and NOTCH2, CDKN2A, HRAS, KRAS, EGFR and the epigenetic regulators such as KMT2C and KMT2D [2,22,24].

Interestingly, recent studies have revealed that UVB exposure can also affect the expression and activity of ncRNAs.

Guo Liu and colleagues found that UVB radiation-induced damage in human keratinocytes led to over-expression of the lncRNA HOTAIR. HOTAIR up-regulation promotes the development of cell damage, while HOTAIR down-regulation reduces UVB-mediated damage. Probably, following the damage, HOTAIR up-regulates double-stranded RNA-dependent protein kinase (PKR) expression, activating the PI3K/AKT and NF-kB pathway and promoting the inflammatory process. However, the mechanisms underlying the regulation of HOTAIR expression after UV exposure are still unknown [39].

Although there are no data available in the literature, Jamie J. Bernard et al. have supposed that in keratinocytes, the UVB irradiation induces alterations in the double-stranded domains of some ncRNAs. These damaged ncRNAs are released into the microenvironment and stimulate the inflammatory response with the production of tumor necrosis factor α (TNF-α) and interleukin-6 (IL -6) in non-irradiated keratinocytes and peripheral blood mononuclear cells (PBMCs), increasing the extent of the damage and the development of cancer lesions [40].

## 3. Role of ncRNAs in CSCC

**Figure 1:**
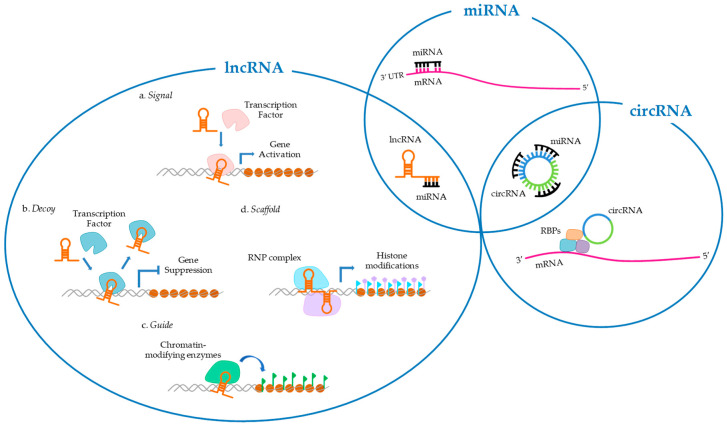
Overview of the interactions and functions of ncRNAs in CSCC. ncRNAs are involved in several fundamental cellular processes, such as cell proliferation, apoptosis, migration, invasiveness, and angiogenesis through chromatin remodelling, transcription, post-transcriptional modifications, and signal transduction. Specific activities of ncRNAs are limited in separated circles, while common involvements are reported in the overlapping circles. miRNAs can target mRNAs on the 3′ UTR site by inhibiting gene expression or circRNAs and lncRNAs by regulating their stability. In turn, circRNAs can directly regulate the gene transcription of mRNAs by binding and sequestering RNA-binding proteins (RBP) with the formation of the RNA-protein complex (RPC). In addition, both circRNAs (CDR1as, circ_0070934 [42]) and lncRNAs (HOTAIR [43],LINC00319 [46], LINC00963 [47]) can sequester miRNAs by acting as “miRNA sponges” in order to regulate their availability. lncRNAs are classified into four categories according to their functions: (a) as signals, helping genes transcription; (b) as decoys, binding and removing transcription factors from chromatin in order to suppress gene expression; (c) as guides, acting, either in cis or trans, on target genes and promoting their activation or repression through the recruitment of chromatin-modifying enzymes; (d) as scaffolds, forming a ribonucleoprotein (RNP) complex that affects histones modifications (MALAT [38]).

Only about 2% of the whole human genome consists of protein-coding gene sequences, while the majority of the remaining genomic sequences transcribe into non-protein-coding DNA that includes structural RNAs (rRNA and tRNAs) and regulatory RNAs (scRNA, miRNA, snoRNA, lncRNA) [41]. Specifically, ncRNAs play a crucial role as mediators in regulating cell processes, despite being considered, only a few decades ago, nothing more than “junk” transcription products. ncRNAs consist of a wide range of non-coding transcripts, including miRNAs, circRNAs, lncRNAs, small interfering RNAs (siRNAs), piwi-interacting RNAs (piRNAs), small nuclear RNAs (snRNAs), and small nucleolar RNAs (snoRNAs) [15]. These RNA molecules can regulate several cellular processes by interacting directly with each other. For example, miRNAs can target mRNAs by inhibiting gene expression, or circRNAs and lncRNAs by regulating their stability [15,16]. On the other hand, circRNAs and lncRNAs can sequester miRNAs by acting as “sponges” to regulate their availability [42,43]. Furthermore, ncRNAs can regulate cellular physiological functions by interacting also with DNA and proteins [15,44,45] (Figure 1). The aberrant alterations of these networks can lead to the development of pathological cell conditions, including tumorigenesis. To date, recent studies have identified several miRNAs, circRNAs, and lncRNAs involved in CSCC, although their mechanisms of action are still unclear, while the role of siRNA, piRNA, snRNA, and snoRNA in these non-melanoma skin cancer remains still unexplored [15].

### 3.1. miRNAs

miRNAs are a large class of single-strand, small (22–24 nt), non-coding transcripts that are produced naturally in animal cells by cleavage of the larger precursor with a stem-loop structure (pri-miRNA) that may contain more than one miRNA sequence. Mature miRNAs are obtained after a two-step process that involves an initial cut by a nuclear RNAse III called Drosha obtaining a pre-miRNA, and a second cut within the stem in the cytoplasm by another RNase III called Dicer [48]. Mature miRNAs post-transcriptionally regulate the expression of several genes by binding to complementary sequences in the coding or in the 3’-untranslated region (3’-UTRs) of target mRNAs. They can control the gene expression in two ways: (i) by degradation of mRNAs when they base-paired perfectly to their target molecule; (ii) by inhibition of protein production if the base-pairing is not perfect [16,49]. In this way, miRNAs can activate as well repress translation, regulating many cellular processes such as cell growth, proliferation, migration, and apoptosis [49]. Furthermore, miRNAs are also involved in regulating the expression of cancer-related genes by promoting the initiation, development, invasiveness, and aggressiveness of several tumors, including CSCC [50]. miRNAs could act as oncogenes (oncomiRs) or tumour suppressors, depending on the roles of their target genes [16]. OncomiRs are often significantly up-regulated in CSCC compared to normal skin, both in tissues and in cell lines, while the tumor suppressor miRNAs are down-regulated and their over-expression can inhibit the CSCC progression [51]. Therefore, miRNAs could be considered as prognostic potential biomarkers and new therapeutic candidates in CSCC target therapy [50].

In the last few years, several published reviews have shown a wide range of miRNAs that play a pivotal role in CSCC tumorigenesis. In Table 2, a summary of the well-known miRNAs involved in the CSCC progression and in drug response is reported. 

Moreover, recent studies have discovered novel oncomiRs (miR-506 and miR-664) and tumor suppressor miRNAs (miR-216b, miR-125b, and miR-3619-5p) involved in the pathogenesis of CSCC, and understanding their role as biomarkers and potential targets for the treatment of CSCC is of great interest in oncology research.

#### 3.1.1. miR-506

Nowadays, preliminary investigations have shown the up-regulation of miR-506 in both CSCC tissues and cell lines compared to surrounding normal tissues and healthy skin cells, but its functional role is not yet well-known. Molecular assays have revealed that the silencing of miR-506 reduces cell proliferation, invasion, and migration ability in CSCC cells, suggesting its involvement in regulating cell survival and apoptosis. miR-506 directly targets the 3′-UTR of Laminin C1 (LAMC1) and nuclear factor NF-kappa-B p65 subunit (p65) by down-regulating their expression. Therefore, miR-506 induces both LAMC1 silencing by preventing cell adhesion and promoting CSCC progression and invasiveness and p65 silencing by inhibiting NF-κB(p65) signaling pathway-mediated apoptosis. These findings have demonstrated that miR-506 acts as an oncomiR and might be a good candidate as a new diagnostic and therapeutic biomarker in CSCC [63].

#### 3.1.2. miR-664

Aberrant expression of the miR-664 has been observed in several cancer types, including CSCC. miR-664 is up-regulated in CSCC cells and tissues, determining an increased cell proliferation, migration, and invasiveness in vitro, and enhanced tumor growth in the xenograft mouse model. It acts as an oncogene by targeting the 3′ UTR of Interferon Regulatory Factor 2 (IRF2) mRNA, a transcriptional factor with anti-tumoral functions in CSCC cells. IRF2 is inhibited by miR-664 and its expression in CSCC is strongly reduced compared to healthy skin tissues [65].

#### 3.1.3. miR-216b

The tumor suppressor miR-216b has been recently observed in CSCC. Its down-regulation in CSCC cells and tissues is related to the over-expression of the Targeting Protein for Xenopuskinesin-like protein 2 (TPX2), a microtubule-associated protein involved in the cell cycle and capable of promoting tumor growth and progression [92]. Further investigations have also shown a correlation between miR-216b and p53 expression, suggesting that the over-expression of miRNA-216b can inhibit cell proliferation and promotes cell apoptosis by negatively regulating TPX2 and promoting the activation of p53 signaling [92].

#### 3.1.4. miR-125b

The role of the miR-125b as a tumor suppressor by targeting the 3′UTR of Matrix Metallopeptidase 13 (MMP13) is already well-known, together with its down-regulation in CSCC compared to healthy skin [96]. Recently, KeTian and colleagues [97] have identified the Signal Transducer and Activator of Transcription 3 (STAT3) as a novel mRNA target in CSCC cells and tissues. miR-125b inhibits the tumor growth and progression in two ways: (i) by influencing the STAT3 pathway, and (ii) by negatively regulating the downstream targets Cyclin D1 and Bcl2, involved in the cell cycle and apoptosis processes, respectively. Therefore, miR-125b plays a tumor suppressor role, targeting both MMP13 and STAT3 mRNAs with inhibition of cell proliferation and migration, and induction of cell apoptosis. It has also been reported that the miR-125b-STAT3 axis is involved in the development and homeostasis of different types of cancer [108,109]. In this perspective, a new therapeutic strategy focused either on the activation of miR-125b or inhibition of STAT3 signaling [97] could be strategic for CSCC.

#### 3.1.5. miR-3619-5p

miR-3619-5p acts as a tumor suppressor in several human malignancies, such as bladder carcinoma [110], prostate cancer [111], non-small cell lung cancer [112], including CSCC [17]. A recent study by Mingfeng Zhang and colleagues has shown the key role of miR-3619-5p in inhibiting cell proliferation and increasing the sensibility in cisplatin-resistant CSCC cells. miR-3619-5p targets and suppresses the expression of Karyopherin subunit alpha 4 (Importin Alpha 3, KPNA4), an importin involved in nuclear translocation of numerous transcription factors. It is down-regulated in CSCC cells and its expression seems to be further reduced in cisplatin-resistant CSCC cells. Recently Mingfeng Zhang et al. [17] have up-regulated and silenced the miR-3619-5p in cisplatin-resistant CSCC cells and in parental cells. Their results showed that the over-expression of miR-3619-5p improved the cell sensitivity to cisplatin by down-regulating the expression of KPNA4, while its depletion induced both an increased expression of the miRNA target and stronger resistance to platin-based chemotherapy. Therefore, miR-3619-5p prevents the cell proliferation and restores the cisplatin-sensitivity in CSCC cells by regulating KPNA4 expression. For all these reasons, miR-3619-5p could be a potential therapeutic target in future CSCC treatment [17].

#### 3.1.6. Possible Therapeutic Applications of the Novel OncomiRs and Tumor Suppressor miRNAs in CSCC

In order to suggest possible therapeutic applications of the novel oncomiRs (miR-506 and miR664) and tumor suppressor miRNAs (miR-215b, miR-216b, and miR-3619-5p) in CSCC, we focused on the evidence, reported in other tumor pathologies, on their role in the modulation of response/resistance to drugs used in the treatment of CSCC.

As previously reported, the up-regulated miRNA-506, when silenced, reduces the urge to proliferate and invade of CSCC cells. This has also been found in serous ovarian cancer through the inhibition of the epithelial-to-mesenchymal transition (EMT) [113]. Liu and coauthors also demonstrated that this miRNA sensitized cells to cisplatin through targeting of the RAD51 gene [64]. A similar behavior in lung cancer cells [66] and in cervical cancer [67] has been shown for the up-regulated miRNA-664 that, when up-regulated, increases tumor proliferation and sensitivity to cisplatin through the modulation of E cadherin, vimentin, and Snail expression. Therefore, it could be hypothesized that the limited and unsuccessful efficacy of the platin-based pharmacological treatment may depend on a lack of correct stratification of patients on a genetic basis; probably the subset of patients with high levels of miRNA-506 or miRNA-664 could respond to cisplatin.

Despite the interaction with drugs of the two tumor suppressor miRNAs, miR-216b and miR-125b have already been characterised in several cancer diseases. The reduced levels of miR-216b appear to be responsible for the reduced sensitivity to cisplatin through an up-regulation of PARP-1 in ovarian cancer cells and via c-Jun/Bcl-xl in NSCLC [93,94]. Similar results are also reported for miR-125b, whose lower levels are related to a reduced response to cisplatin in ovarian cancer cells and in nasopharyngeal carcinoma cells affecting the Bcl-2 family [98,99], in thyroid cancer through Foxp3 up-regulation [100] and in NSCLC, osteosarcoma and gastric cancer [101,102,103]. Low levels of miR-125b are also correlated with resistance to 5-FU in colorectal cancer [104] and to doxorubicin in breast cancer [105,106]. Moreover, as demonstrated by Chen, low levels of miR-216b up-regulate Beclin-1 by reducing sensitivity to cetuximab through the induction of autophagy in CRC cells [95]. All these evidence might suggest that even in the CSCC, the low levels of the two miRNAs could be responsible for the resistance to platin-based and anthracyclines-based chemotherapy and that these drug resistance mechanisms could be reversed by inducing their expression using miRNA mimics conveyed with appropriate nanovectors [114]. Furthermore, as reported above, the over-expression of miRNA-3619-5p is involved in drug response in CSCC by reducing KPNA4 expression and increasing cisplatin sensitivity [17].

We have already highlighted how the use of immunotherapy with checkpoint inhibitors (ICI) is considered the gold standard therapy for patients with recurrent or metastatic CSCC [1,9,10].The investigation of the possible role of the selected miRNAs in the response or resistance to ICI, also in other tumors, has shown that only miR-125b can be a predictor of response. Indeed, it is part of a set of miRNAs in plasma which, when present at high levels, correlates with shorter PFS and OS in melanoma patients treated with anti-PD1, nivolumab, and pembrolizumab [107].

### 3.2. Circular RNAs

Among the ncRNAs, the circRNAs present continuous covalently closed circular structures, likely originating from protein-coding genes due to ‘back-splicing’, an aberrant splicing process in which the 5’ splice site is joined to an upstream 3’ splice site. They have an average length of 500 nucleotides and lack free ends that convey a higher resistance against the degradation activity of the nuclease, making them more stable than the linear RNA. There are four main types of circRNAs: (i) exonic circRNAs originating from single or several exons; (ii) circular intronic RNAs (ciRNAs) originating only from introns; (iii) exonic-intronic circRNAs (ElciRNAs) originating either from introns or from exons; (iiii) tRNA intronic circRNAs (tricRNAs) that are generated by a 3′-5′ phosphodiester bond by splicing pre-tRNA intron [115]. The circRNAs’ structural features confer them functional properties in the regulation of transcriptional and post-transcriptional events [45,116], such as: (i) direct regulation of gene transcription by binding and sequestering RNA-binding proteins (RBP) with the formation of RNA-protein complexes (RPCs) which interact with linear RNA [42,45]; (ii) regulation of post-transcriptional events, acting as a ‘miRNA sponge’, by sequestering multiple specific miRNAs per single circRNA with their own miRNA response elements (MREs). Recent evidence has shown that circRNAs is widely expressed in tumor tissues and are involved in the regulation of cancer-related pathways; nevertheless, little is known about their expression and function in the CSCC [42,116]. Mahapatra and colleagues in a recent study have identified 227 and 150 circRNAs in normal skin and CSCC samples, respectively. Along with the best-known expressed circRNAs, such as the Cerebellar Degeneration-Related protein 1 antisense RNA (CDR1as) which targets the tumor suppressor miR-7 [117], they have identified six novel uncharacterized circRNAs derived from IFFO2, PLIN4, DMKN, METRNL, KRT1 and POF1B genes, with an abundance of circ_IFFO2 and circ_PLIN4 in the normal skin [33]. The greatest part of these circRNAs are structurally characterized by two exons, such as those found in other tissues and tumor types [118]. Differential expression analysis showed a strong reduction of circRNAs in CSCC samples, probably related to the over-expression of the circRNA-biogenesis ADAR negative regulator [33,118]. RNA-seq analysis showed the down-regulation of several circRNAs related to epidermal differentiation (circ_MBOAT2, circ_PTPN13 and circ_ACVR2A) in CSCC [118]. In addition, the reduction of both the well-known cancer-associated circRNA CDR1as and the new circRNA circ_IFFO2 in precancerous skin lesions, actinic keratosis (AK) and CSCC samples, has been observed, suggesting their role as initial events in CSCC progression [33]. On the other end, some circRNAs are over-expressed in CSCC compared to normal skin, such as circ_EPSTI that acts on the interferon-response gene (EPSTI1) that has been shown to be involved in tumor progression and invasion in various types of cancer [33]. Xiaoxia An et al. have identified a novel circRNA (circ_0070934) highly expressed in CSCC tissue specimens and cell lines which promotes the proliferation, migration, and invasiveness in CSCC cells by sponging and negatively regulating miR-1238 and miR-1247e5p, two microRNAs that act as tumor suppressors. Therefore, the circ_0070934 plays an oncogenic role by sustaining CSCC progression [116].

These results highlight the direct relationship between circRNAs and tumor progression by supporting circRNAs as future targets in the diagnosis and therapy of CSCC.

#### Possible Therapeutic Applications of circRNAs in CSCC

To date, very little is known about the role of circRNAs (MBOAT2, circ_PTPN13, circ_ACVR2A, circRNA CDR1, circ_IFFO2, circ_EPSTI and circ_0070934) in the response or induction of resistance to drugs used in CSCC or in other tumors. The only exception is a study on the possible contribution of circRNA CDR1to pemetrexed and cisplatin chemoresistence [119]. The authors have reported a high expression of this circRNA in pemetrexed and cisplatin-resistant lung adenocarcinoma tissues, with a chemoresistance that could be related to the EGFR/PI3K signaling pathway. Although there is no robust evidence suggesting an interaction between circRNAs and drugs, their investigation could be useful to investigate this topic in order to identify predictive factors of drug response for a better selection of patients to undergo therapy.

### 3.3. lncRNAs

lncRNAs are identified as a wide and largely uncharacterized group of RNA transcripts longer than 200 nucleotides that cannot be translated into proteins. Their transcription is regulated by mechanisms similar to those of the protein-coding genes; indeed the lncRNA genes are transcribed by the RNA polymerase II, and their transcripts are often spliced, capped at the 5′termini, and polyadenylated at the 3′ termini [120]. Up to now, over 18,000 lncRNA transcripts have been detected in the human genome, and according to their genomic position relative to the nearby protein-coding genes, they are classified into the following categories: intergenic, sense intronic, antisense, and bidirectional lncRNA. Long intergenic RNAs (lincRNAs) are located between two protein-coding genes, usually in enhancer regions, and can induce chromatin modification in the promoters of the downstream genes. Sense intronic lncRNAs are located in intronic regions and are transcribed from the sense strand of protein-coding genes without overlapping exonic sequences. Antisense lncRNAs are transcribed from the antisense strands and can include natural antisense transcripts. Bidirectional lncRNAs have transcription start sites at about 1 kb from the promoter region of a protein-coding gene but are transcribed in the opposite direction [29,121]. lncRNAs have tissue-specific expression and are involved in several cell processes by regulating tissue homeostasis, development, repair, or stress response [28]. Based on their functions, the lncRNAs can be classified into four categories: signal, decoy, guide, and scaffold. Signal lncRNAs are involved in the transmission of signaling pathways, regulating downstream genes transcription; the decoy lncRNA binds and removes transcription factors from chromatin to regulate gene expression; guide lncRNAs can act, either in cis or trans, on target genes by recruiting chromatin-modifying enzymes; scaffold lncRNAs can bind together multiple proteins to form a complex and modify histone in chromatin [3,120]. Therefore, the lncRNAs can interact with DNA, RNA, and proteins.

A growing body of evidence has shown that lncRNAs are involved not only in tissue homeostasis but also in the regulation of tumor initiation, progression, and metastasis with either tumor-promoting or tumor-suppressive functions. Nonetheless, the role of lncRNAs in the cutaneous biology and CSCC tumorigenesis is not yet well known [28,33]. The analysis of the lncRNA landscape reveals their different expression in normal skin and in CSCC samples, in according to the above-mentioned tissues-specific expression of lncRNAs. Via recent transcriptomic studies, Mahapatra and co-authors have identified about 908 lncRNAs differentially expressed in CSCC samples compared to normal skin, of which 319 are up-regulated and 589 down-regulated [33]. In CSCC, these lncRNAs can perform oncogenic (e.g., MALAT1 and LINC00319) or suppressive (e.g., TINCR and LINC00520) functions by regulating transcriptor factors activity and mRNA stability directly or indirectly by interacting with microRNAs [23]. This evidence highlights the pivotal and complex role of lncRNAs in CSCC progression and suggests them as putative biomarkers and therapeutic targets in human CSCC. In Table 3, the lncRNAs involved in CSCC described below are reported.

#### 3.3.1. MALAT1

Metastasis-associated lung adenocarcinoma transcript 1 (MALAT1) is a highly expressed lncRNA in many solid tumors and promotes cancer progression by regulating the alternative splicing and gene transcription [147]. MALAT1 is widely expressed in CSCC, but its role in this cutaneous cancer is not yet well-known. Recently, Zhang Y. and co-authors have explored the oncogenic role of MALAT1 in CSCC, demonstrating that MALAT1 knockdown strongly reduces proliferation, migration, invasiveness, and increases apoptosis. MALAT1 sustains its tumorigenic role by up-regulating EGFR, a transmembrane receptor that contributes to cell growth and proliferation by activating the MAPK and PI3K pathways. Transcriptomic sequencing has revealed the crucial role of kinectin 1 (KTN1) as a mediator in the up-regulation of EGFR protein expression by MALAT1. In addition, a specific RNA-protein interaction has been identified between MALAT1 and c-MYC, a common regulator of cancerogenesis in many tumors. MALAT1 and c-MYC form a complex that directly binds to the promoter region of the KNT1 gene, enhancing its activation and, in turn, positively regulating EGFR gene transcription. Mechanistically, these interactions establish a novel c-MYC-assisted MALAT1-KTN1-EGFR axis which promotes tumor development and which might be considered as an innovative therapeutic target in CSCC [38].

#### 3.3.2. TINCR

Tissue differentiation-inducing non-protein-coding (TINCR) is a lncRNA involved in the post-transcriptional regulation of human epidermal differentiation and in the maintenance of the normal structure and function of skin. TINCR is down-regulated in CSCC compared to normal skin and the lower availability of TINCR contributes to the dysregulation of cutaneous cell differentiation and supports CSCC tumorigenesis. Recent evidence suggests that TINCR may be involved in ALA-PDT-induced (5-aminolevulinic acid- photodynamic therapy-induced) apoptosis and autophagy in CSCC cells. ALA-PDT is a non-invasive therapeutic strategy widely used in dermatological oncology for the treatment of premalignant and malignant skin lesions. Via photochemical and photobiological reaction, ALA-PDT generates reactive oxygen species (ROS) which can induce cell death and promote the inhibition of CSCC progression [146]. ROS especially leads to the activation of the ERK1/2 pathway, which actives the transcription factor SP3 (specificity protein 3) through direct phosphorylation [148]. Interestingly, SP3 binds the TINCR promoter and supports its transcription. Therefore, ALA-PDT can induce TINCR expression in CSCC cells through the ERK1/2-SP3 pathway via ROS production and TINCR over-expression that supports the apoptosis and cell autophagy in CSCC. This evidence highlights the crucial role of lncRNA TINCR in supporting the local therapeutic strategy [146].

#### 3.3.3. THOR

Testis-associated Highly-conserved Oncogenic (THOR) is a lncRNA highly expressed in testis and in a broad range of human cancers. THOR over-expression contributes to cancer progression since its silencing or depletion is well-known to induce the inhibition of cancer cell proliferation. At the molecular level, THOR exhibits a conserved interaction with Insulin-like growth factor 2 mRNA-binding protein 1 (IGF2BP1), an RNA-binding protein with a potential oncogenic role in CSCC [131,149]. Recent genomic and proteomic analyses have revealed a strong expression of both THOR and IGF2BP1 in primary human CSCC cells and tissues. Mechanistically, it has been observed that THOR knockdown in CSCC cells induces the down-regulation of IGF2BP1 mRNA, which is correlated with the inhibition of the progression of cutaneous cancer [131]. Therefore, this evidence suggests the great importance of THOR-IGF2BP1 interaction, supporting THOR as a novel potential target in CSCC therapy.

#### 3.3.4. HOTAIR

HOX antisense intergenic RNA (HOTAIR) is a lncRNA involved in different processes of normal cell development, but its aberrant over-expression can contribute to proliferation, invasion, and infiltration of malignant tumor cells [150,151]. qRT-PCR assays on CSCC cells and normal human keratinocytes have shown an increased HOTAIR expression in CSCC cells, suggesting its involvement in cutaneous pathogenesis. Further investigations have highlighted the correlation between higher expression of HOTAIR and enhanced proliferation and migration ability of CSCC cells in vitro, and an increased tumor growth in vivo, otherwise attenuated by the HOTAIR silencing. In a recent study, Guo-Jun Yu and co-authors explored the mechanisms underlying HOTAIR involvement in CSCC occurrence. It has been seen that the lncRNA HOTAIR functions as a competitive endogenous RNA (ceRNA) to regulate the Prenylated Rab acceptor 1 domain family, member 2 (PRAF2) expression, by sponging miR-326 in CSCC cells. PRAF2 is a miRNA-326 target gene closely related to cancer progression and widely up-regulated in the CSCC. These results support the HOTAIR/miR-326/PRAF2 axis in CSCC in which the binding HOTAIR/miR-326 contributes to CSCC occurrence and development, with a positive modulation of the PRAF2 expression [43]. Further insights are needed to understand the role of HOTAIR as a potential biomarker and therapeutic target for CSCC.

#### 3.3.5. LINC00520

The long intergenic non-protein coding RNA 520 (LINC00520) plays a key role in cell migration, invasion, and metastasis in breast cancer [152]. Recent evidence shows that LINC00520 is expressed in CSCC, carrying out an anti-proliferative activity [37]. It is well-known that in CSCC carcinogenesis, the aberrant activation of the EGFR gene leads to higher activation of the PI3K-Akt anti-apoptotic signaling pathway, which contributes to cancer progression [35]. The latest studies show that EGFR is a target gene of LINC00520. Therefore, LINC00520-mediated inhibition of EGFR suppresses the PI3K/AKT signaling pathway by preventing the progression, invasiveness, and migration of CSCC cells in vitro and by reducing tumor growth and metastasis in vivo [37].These findings suggest LINC00520 as a new potential therapeutic target for novel therapeutic approaches in the CSCC.

#### 3.3.6. LINC00319

Cancer-related long intergenic non-protein coding RNA 00319 (LINC00319) is considered a cancer promoter in several human cancers such as lung [153], nasopharyngeal [154], and ovarian cancer [155], but its role in CSCC still remains unclear. LINC00319 is widely up-regulated in CSCC cells and tissues, promoting cell proliferation, migration, invasiveness, and the inhibition of apoptosis. Functional studies show that LINC00319 carries out its pro-tumoral function as a ceRNA interacting with miR-1207-5p, thereby regulating the cyclin-dependent kinase 3 (CDK3) expression, a miR-1207-5 p target gene. CDK3, an ATP-dependent serine/threonine kinase, regulates the cell cycle progression by promoting the transition from the G0/G1 to G1/S phase. These results highlight the positive correlation between the over-expression of LINC00319 and CDK3 up-regulation in CSCC and thus suggest LINC00319 involvement in the CSCC progression. Based on this evidence, LINC00319 could be considered an eligible target for the CSCC pharmacological strategies [46].

#### 3.3.7. LINC00963

Long intergenic coding RNA 963 (LINC00963) plays a carcinogenic role in several cancers, such as non-small cell lung cancer [156], hepatocellular carcinoma [157], and melanoma [158]. It has been reported that LINC00963 is significantly up-regulated in CSCC cell lines compared to the normal skin. Functional studies have revealed that LINC00963 regulates cell proliferation and migration in CSCC by miR-1193/SOX4 axis: LINC00963 binds miR-1193 and down-regulates the microRNA expression. In turn, miR-1193 negatively regulates the expression of SOX4, a transcription factor, belonging to the SOX family, involved in tumor progression and invasiveness. However, in CSCC cells, SOX4 appears over-expressed at mRNA and protein levels. Mechanistically, LINC00963, suppressing miR-1193 expression promotes SOX4 up-regulation and cancer progression. These findings confirm the role of theLINC00963/miR-1193/SOX4 axis in the CSCC tumor progression, proposing this lncRNA as a promising biological target [47]. Since the LINC00963/miR-1193/SOX4 axis has only been explored in CSCC cell lines, further investigations in patient-derived CSCC models are warranted to identify the clinical significance of this lncRNA/microRNA/gene correlation.

#### 3.3.8. LINC01048

Long intergenic non-protein coding RNA 1048 (LINC01048) is widely expressed in CSCC samples and represents a poor prognostic factor for CSCC patients. The analysis of survival curves by the Kaplan–Meier method has revealed a lower overall survival and disease-free survival in patients with higher LINC01048 levels. These data suggest LINC01048 involvement in cutaneous cancer progression. A recent study on CSCC patient-derived samples has revealed that LINC01048 plays a key role in the regulation of the Hippo pathway leading to the up-regulation of the YAP1 oncogene transcription. Notably, the lncRNA can regulate gene expression by interacting with RNA-binding proteins. It promotes the transcription activation of YAP1 by binding to the transcription factor TAF15, an RNA-binding protein. Therefore, in CSCC, the LINC01048-mediated YAP1 over-expression is the key-driver of cell proliferation. Furthermore, functional studies have demonstrated that LINC01048 can promote cell proliferation by preventing cell apoptosis through the regulation of the expression of Bax and Bcl-2, two apoptosis-related proteins. This evidence supports the prognostic value of LINC01048 in CSCC and its role as a potential therapeutic biomarker [143].

#### 3.3.9. LINC00162 or PICSAR

Long intergenic non-protein coding RNA 162 (LINC00162) is highly expressed in tumor CSCC cells and appears to be the most up-regulated lncRNA among those identified. Its expression is regulated by the p38MAPK pathway, especially by p38α and p38δ isoforms; for this reason, it has been designed as P38 Inhibited Cutaneous Squamous cell carcinoma Associated lincRNA (PICSAR). RNA-ISH analysis of broad tissue samples derived from normal skin, premalignant lesions, and CSCC has revealed the tumor-specific expression of PICSAR, suggesting the importance of this lncRNA as a prognostic marker for CSCC [29]. Furthermore, it has been found that PICSAR is involved in the regulation of different cell processes. It is well-known that UVA radiations play a key role in cutaneous carcinogenesis by promoting an over-activation of ERK1/2 signaling in keratinocytes [159], but UVA radiations are not the only factor implicated in MAPK/ERK pathway dysregulation in CSCC. The lncRNA PICSAR down-regulates the dual-specificity phosphatase 6 (DUSP6), a specific ERK2 phosphatase, both at mRNA and protein level. As result, the lower expression of ERK2′s negative regulator increases ERK1/2 activation in CSCC cells by supporting cell growth and proliferation [29]. In addition, PICSAR promotes cell migration by down-regulating the expression of α2β1 and α5β1 integrin on the cell plasma membrane by preventing the cell adhesion on collagen I and fibronectin [144]. This evidence highlights the pivotal role of lncRNA PICSAR in the carcinogenesis of CSCC, and therefore, it may be considered as a biomarker for the progression of CSCC and a potential therapeutic target in these non-melanoma skin cancer [29].

#### 3.3.10. LINC00346 or PRECSIT

A recent study on lncRNAs in CSCC has revealed the over-expression of the long intergenic non-protein coding RNA 346 (LINC00346) in CSCC samples compared to normal skin, suggesting its involvement in cutaneous carcinogenesis. LINC00346 expression is regulated by p53; the loss of function of the p53 gene, an early common event in CSCC carcinogenesis, indirectly up-regulates the expression of LINC00346. RNA-seq and proteomic analysis have demonstrated that LINC00346 is involved in the regulation of STAT3, a transcriptional activator of several genes, such as matrix metalloproteinase (MMP) genes, both at mRNA and protein level. In CSCC, LINC00346 up-regulates the expression of STAT3, which in turn induces an increased expression of matrix metalloproteinase MMP-1, MMP-3, MMP-10, and MMP-13, which are notably involved in cell migration and invasion. Therefore, LINC00346 through the targeting of STAT3 signaling can contribute to CSCC progression. Based on this evidence, the lncRNA LINC00346 has been named p53 regulated carcinoma-associated STAT3-activating long intergenic non-protein coding transcript (PRECSIT), and can be regarded as a new eligible therapeutic target in CSCC [28].

#### 3.3.11. Possible Therapeutic Applications of lncRNAs in CSCC

“The role of the lncRNAs” reported in Table 3 in the response and resistance to drugs used for the treatment of CSCC patients has been extensively studied in various tumor pathologies. A brief summary of the most significant pieces of evidence is reported below, suggesting that, even in CSCC, these ncRNAs could play a role as predicting factors or therapeutic targets.

MALAT1 is elevated in CSCC and appears to play an oncogene role through the activation of the EGFR pathway [38], as already demonstrated in various tumor pathologies, such as lung cancer, laryngeal squamous cell carcinoma, ovarian cancer, bladder cancer, gastric cancer, and oral squamous cell carcinoma, in which it is associated with a poor response to cisplatin through various mechanisms such as the axis MALAT1/miR-101/SOX9, MALAT1/miR-101-3p/MCL1 and MALAT1/miR-101-3p/VEGF-C or the upregulation of multi-drug resistant proteins (MDR) and the PI3K7AKT pathway [122,123,124,125,126,127,128]. Since this lncRNA has been shown to be down-regulated by cisplatin in the laryngeal squamous cell carcinoma model [160], it could be a promising predictor of response to platin-based chemotherapy in CSCC if the evidence reported by Chen is confirmed in this pathology, too [160]. For lncRNA THOR, as well as for LINC00346, there is only one piece of evidence for each which demonstrates that their high expression is linked to a reduced sensitivity to cisplatin due to increased cell stemness for THOR and the inhibition of miR-342-5p for LINC00346 [132,145]. For lncRNA HOTAIR, similar evidence is reported in various tumor pathologies, such as lung cancer via p21 down-regulation and ovarian cancer, by activating the Wnt/b-catenin pathway or inhibiting EZH2 and SIRT1 [133,134,135,136]. There is no evidence in the literature on the interaction between cisplatin and the other lncRNAs with the role of oncogene reported in Table 3. In conclusion, all these pieces of evidence suggest that the described lncRNAs may be responsible for the reduced response to platinum derivatives, and therefore could be considered as therapeutic targets to restore sensitivity to these chemotherapeutics by using tumor-specific peptides [161].

Regarding 5-FU, MALAT1 seems to mediate the resistance by inducing the expression of MDR proteins in colorectal cancer cells and by inhibiting apoptosis through the modulation of multiple pathways in the HCC cell lines [129,130]. Resistance to 5-FU could be also mediated by HOTAIR in *in vitro* models of esophageal cancer and of colorectal cancer [137,138] and when given in combination with oxaliplatin in gastric cancer [139]. Also, for 5-FU, no interaction with other lncRNAs is reported.

Resistance to doxorubicin treatment is showed to be correlated only with HOTAIR in various tumor pathologies, such as gastric cancer and bladder transient cell carcinoma [140,141], and cells whose down-regulation restores the sensitivity in breast cancer cell lines through PI3K/Akt pathway modulation [142].

Finally, there are no studies in the literature that report the possible role of these lncRNAs in the response or resistance to cemiplimab and pembrolizumab used as immunotherapy in CSCC.

## 4. Conclusions

In summary, this review provides an overview of the mutational landscape of CSCC mainly induced by prolonged UVR exposure and on the impact of the high mutation burden in promoting tumor initiation, growth, and proliferation. In this context, we also exhaustively explore the role of both well-known and previously uncharacterized ncRNA transcripts in the regulation of pivotal intracellular signaling pathways as oncogenes or tumor suppressors.

Furthermore, we reported how these ncRNAs impact on the chemosensitivity and chemoresistance in tumors other than CSCC in order to demonstrate that their characterization in this common non-melanoma skin cancer could provide new insights to optimize therapeutic approaches.

In fact, although immunotherapy has revolutionized the therapeutic approach of advanced CSCC, about half of patients do not respond to this therapy, and their prognosis is particularly severe with a survival time of only a few months. To date, it is not possible to select patients who have a real clinical benefit from this therapy from those who do not benefit from it. Thus, it is our opinion that an appropriate selection of patients on a genetic basis, also considering ncRNAs as well as the mutational burden, could allow better responses to chemotherapy treatments and immunotherapy.

The other pressing clinical need is the search for new treatments for patients who are non-responders to ICIs and for those who relapse after a more or less long response. Several preclinical studies for the evaluation of novel anticancer strategies by targeting oncogenic ncRNAs [www.clinicaltrials.gov] demonstrate how targeting ncRNAs in cancers is a promising strategy and could represent a novel pharmacological approach in CSCC treatment. However, it is necessary to take into account the low bioavailability of these nucleic acid drugs in vivo, RNA degradation and instability, and off-target effects. So, the development of advanced ncRNAs delivery strategies through nanotechnological approaches that provide novel ncRNA carriers or systems ensuring minimal off-target effects and toxicity is urgently needed.

In conclusion, the identification of functional miRNAs, circRNAs, and lncRNAs in the CSCC and the understanding of their molecular networks can provide: (i) new promising prognostic factors that would allow us to evaluate the aggressiveness of this common non-melanoma skin cancer, (ii) novel predictive factors of chemoresistance and chemosensitivity, and (iii) new potential pharmacological targets whose suppression or up-regulation can be used as a future therapeutic approach in CSCC.

## Figures and Tables

**Table 1:** The most common UV-induced gene mutations in CSCC.

Gene	Transation	Mutation Rate in CSCC	Mechanisms	ncRNA Target	Ref.
TP53	C > T transition	~50–90%	UVR inactivates TP53 gene and down-regulates its expression, promoting pro-survival, pro-invasive and pro-tumorigenic functions in CSCC.	- miR-216b - lncRNALINC00346	[2,23,25,26,27,28]
NOTCH1 NOTCH2	G > A transition	~82%	UVR induces loss of function of NOTCH1 and NOTCH2 genes and down-regulates their expression, sustaining tumor progression.	Not mentioned	[2,22,23,29,30]
CDKN2A	Different nucleotide transitions	~28–50%	UVR inactivates CDKN2A gene leading to uncontrolled cell proliferation.	Not mentioned	[2,22,31,32]
RAS	Different nucleotide transitions at codon 61	~21% (with prevalence of mutation in HRSA).	UVR induces activating mutation that drive the tumorigenic processes by activating the RAF/MEK/ERK1/ERK2 signaling pathway.	Not mentioned	[29,33,34]
EGFR	/	~2.5–3%	UVR-mediated gain-of-function mutations promoting the over-activation of the RAF/MEK/ERK1/ERK2 signaling pathway.	- lncRNA MALAT1 - lncRNA LINC00520 - lncRNALINC00162	[29,33,34,35,36,37,38]
KMT2C KMT2D	/	~67%	UVR inactivates KMT2C gene leading to aggressive CSCC and bone metastasis.	Not mentioned	[24,26]

**Table 2:** Up-regulated (in bold) and down-regulated (normal letters) miRNAs in CSCC.

miRNAs (Function)	Gene Targets	Mechanisms	Drug Sensitivity (S)/Drug Resistance(R) in Other Cancer Models
**miRNA-221** (Oncogene)	3′UTR of PTEN	Regulation of cancer progression by inhibiting the tumor suppressor PTEN [49].	(R) Doxorubicin [52]
**miRNA-142-5p** (Oncogene)	3′UTR of PTEN	Induction of the cancer stem cell-like properties of CSCC through inhibition of PTEN [53].	No data available
**miRNA-365** (Oncogene)	3′UTR of NFIB and BAX	Increase of the tumor development and inhibition of the apoptosis by down-regulating NFIB and BAX expression, respectively [54,55].	No data available
**miRNA-186** (Oncogene)	3′UTR of APAF1 and RETREG1	Promotion of cell proliferation and inhibition of cell apoptosis by negatively regulating APAF1 and RETREG1 expression [56,57].	No data available
**miRNA-31** (Oncogene)	3′UTR of RhoBTB1	Increase of cell proliferation by reducing the expression of the tumor suppression RhoBTB1 [58].	(R) 5-FU [59]
**miRNA-135b** (Oncogene)	3′UTR of LZTS1	Induction of tumor growth, cell motility and invasiveness by down-regulating the expression of the tumor suppressor LZTS1 [60].	No data available
**miRNA-205** (Oncogene)	Not mentioned	Unknown mechanism, even if its up-regulation correlates with local recurrences and bad prognosis [61].	(S) Doxorubicin [62]
**miRNA-506** (Oncogene)	3′UTR of LAMC1 and NF-kB (65)	Down-regulation of LAMC1 and NF-kB (65) expression by promoting the progression, invasiveness and inhibition of apoptosis [63].	(S) Cisplatin [64]
**miRNA-664** (Oncogene)	3′UTR of IRF2	Inhibition of IRF2 expression inducing cell proliferation, migration, and invasiveness in vitro, and an increased tumor growth in the xenograft mouse model [65].	(S) Cisplatin [66,67]
**miRNA-21** (Oncogene)	3′UTR of GRHL3	Reduction of PTEN expression and the activation of PI3K/AKT/mTOR signaling [68].	(R) Cisplatin [69] (R) 5-FU [70] (R) Doxorubicin [71]
miRNA-34a (Tumor suppressor)	3′UTR of HMGB1	Inhibition of cell proliferation, migration and invasion by targeting the nuclear DNA-binding protein HMGB1 [72].	(S) Cisplatin [73] (S) Doxorubicin [74]
miRNA-181a (Tumor suppressor)	3′UTR of KRAS	Cancer development through KRAS signaling [75].	(R) Doxorubicin [76]
miRNA-148a (Tumor suppressor)	3′UTR of MAP3K4 and MAP3K9	Suppression of the MEK pathway by negatively regulation of MAP3K4 and MAP3K9 expression [77].	No data available
miRNA-20a (Tumor suppressor)	3′UTR of LIMK1	Suppression of proliferation, migration and invasion of cancer cells by inhibiting LIMK1 expression. The low miRNA-20a expression correlates with poor prognosis [78,79].	(S) Cisplatin [80] (S) Doxorubicin [80]
miRNA-204 (Tumor suppressor)	3’ UTR of PTPN11	Regulation of the cancer-involved STAT3 pathway targeting of PTPN11 [51].	No data available
miRNA-203 (Tumor suppressor)	3′UTR of c-MYC	Inhibition of cell proliferation, migration and angiogenesis by suppressing c-MYC expression [81].	(R) Cisplatin [82] (S) 5-FU [83]
miRNA-199a (Tumor suppressor)	3′UTR of CD44	Reduction of the CSCC cell proliferation and migration by regulating the expression of CD44 and its interaction with Ezrin [84].	No data available
miRNA-124 (Tumor suppressor)	3′UTR of ERK1	Control of tumor growth by regulating the expression of ERK1 [85].	(S) Cisplatin [86]
miRNA-214 (Tumor suppressor)	3′UTR of ERK1 and ERK2	Regulation of the cell proliferation by modulating ERK2 expression [85].	(S) Cisplatin [87]
miRNA-497 (Tumor suppressor)	3′UTR SERPINE1	Inhibition of the epithelial–mesenchymal transition (EMT) and CSCC cell invasion and metastasis by targeting the Plasminogen Activator Inhibitor-1 (PAI-1), also known as SERPINE1 [88].	5-FU [89]
miRNA-193b-3p miRNA-365a-3p (Tumor suppressor)	3′ UTR of KRAS and MAX	Anti-proliferative and anti-migratory properties by interacting with oncogene KRAS and MYC-associated factor X (MAX) [90].	No data available
miRNA-361-5p (Tumor suppressor)	3′UTR of VEGFA	Inhibition of tumor growth by modulating the expression of the tumor angiogenesis factor VEGFA [91].	No data available
miRNA-216b (Tumor suppressor)	3′ UTR of TPX2	Reduction of cell proliferation and promotion of apoptosis by regulating TPX2 expression [92].	(R) Cisplatin [93,94] (R) Cetuximab [95]
miRNA-125b (Tumor suppressor)	3′UTR of MMP13 and STAT3	Inhibition of the tumor growth and progression by modulating the STAT3 pathway and negative regulation of the downstream targets Cyclin D1 and Bcl2, involved in the cell cycle and apoptosis processes, respectively [96,97].	(R) Cisplatin [98,99,100,101,102,103] (R) 5-FU [104] (R) Doxorubicin [105,106] (R) Pembrolizumab [107]
miRNA-3619-5p (Tumor suppressor)	3′ UTR of KPNA4	Suppression of the KPNA4 expression cell proliferation and increasing the sensibility in CSCC cisplatin-resistant cells [17].	(S) Cisplatin [17]

**Table 3:** Up-regulated (in bold) and down-regulated (normal letters) lncRNAs in CSCC.

lncRNAs (Function)	Gene Targets	Mechanisms	Drug Sensitivity (S)/Drug Resistance (R) in Other Cancer Models
**MALAT1** (Oncogene)	c-MYC gene	Induction of tumor progression by up-regulating EGFR expression and activating the MAPK, PI3K and KRAS pathway [38].	(R) Cisplatin [122,123,124,125,126,127,128] (R)5-FU [129,130]
**THOR** (Oncogene)	IGF2BP1 gene	Induction of cancer progression by targeting the RNA-binding protein IGF2BP1 [131].	(R)Cisplatin [132]
**HOTAIR** (Oncogene)	miR-326	Promotion of cell proliferation and migration in vitro and tumor growth *in vivo* by up-regulating PRAF2 expression by sponging miR-326 [43].	(R) Cisplatin [133,134,135,136] (R) 5-FU [137,138,139] (R) Doxorubicin [140,141,142]
**LINC00319** (Oncogene)	miR-1207-5p	Promotion of cell proliferation, migration, invasiveness and the inhibition of apoptosis by up-regulating the expression of CDK3, a miR-1207-5 p target gene [46].	No data available
**LINC00963** (Oncogene)	miR-1193	Inhibition of miR-1193 expression by promoting SOX4 expression and cancer progression [47].	No data available
**LINC01048** (Oncogene)	Transcription factor TAF15	Induction of cell proliferation by up-regulating YAP1 oncogene transcription and modulating the apoptosis-related proteins expression [143].	No data available
**LINC00162 or PICSAR** (Oncogene)	DUSP6, α2β1 and α5β1 integrin gene	Increase of cell proliferation and migration by down-regulating both the expression of a specific ERK2 phosphatase (DUSP6) and of the α2β1 and α5β1 integrins [29,144].	No data available
**LINC00346 or PRECSIT** (Oncogene)	Transcription factor STAT3	Induction of cell migration and invasion by up-regulating MMP-1, MMP-3, MMP-10 and MMP-13 expression [28].	(R) Cisplatin[145]
TINCR (Tumor suppressor)	Not mentioned	Induction of apoptosis and cell autophagy [146].	No data available
LINC00520 (Tumor suppressor)	EGFR gene	Inhibition of EGFR expression by inactivating the PI3K-Akt pathway and preventing the tumor progression [37].	No data available
